# Differentially Expressed Gene Pathways in the Conjunctiva of Sjögren Syndrome Keratoconjunctivitis Sicca

**DOI:** 10.3389/fimmu.2021.702755

**Published:** 2021-07-19

**Authors:** Cintia S. de Paiva, Claudia M. Trujillo-Vargas, Laura Schaefer, Zhiyuan Yu, Robert A. Britton, Stephen C. Pflugfelder

**Affiliations:** ^1^ Department of Ophthalmology, Baylor College of Medicine, Houston, TX, United States; ^2^ Center for Metagenomics and Microbiome Research, Department of Molecular Virology and Microbiology, Baylor College of Medicine, Houston, TX, United States; ^3^ Grupo de Inmunodeficiencias Primarias, Facultad de Medicina, Universidad de Antioquia UdeA, Medellín, Colombia

**Keywords:** Sjögren syndrome, dry eye, gene expression, conjunctiva, immune pathways

## Abstract

Sjögren syndrome (SS) is an autoimmune condition that targets the salivary and lacrimal glands, with cardinal clinical signs of dry eye (keratoconjunctivitis sicca, KCS) and dry mouth. The conjunctiva of SS patients is often infiltrated by immune cells that participate in the induction and maintenance of local inflammation. The purpose of this study was to investigate immune-related molecular pathways activated in the conjunctiva of SS patients. Female SS patients (n=7) and controls (n=19) completed a series of oral, ocular surface exams. Symptom severity scores were evaluated using validated questionnaires (OSDI and SANDE). All patients fulfilled the ACR/EULAR criteria for SS and the criteria for KCS. Fluorescein and lissamine green dye staining evaluated tear-break-up time (TBUT), corneal and conjunctival disease, respectively. Impression cytology of the temporal bulbar conjunctiva was performed to collect cells lysed and subjected to gene expression analysis using the NanoString Immunology Panel. 53/594 differentially expressed genes (DEGs) were observed between SS and healthy controls; 49 DEGs were upregulated, and 4 were downregulated (*TRAF5, TGFBI, KLRAP1*, and *CMKLRI*). The top 10 DEGs in descending order were *BST2, IFITM1, LAMP3, CXCL1, IL19, CFB, LY96, MX1, IL4R, CDKN1A*. Twenty pathways had a global significance score greater or equal to 2. Spearman correlations showed that 29/49 upregulated DEGs correlated with either TBUT (inverse) or OSDI or conjunctival staining score (positive correlations). Venn diagrams identified that 26/29 DEGs correlated with TBUT, 5/26 DEGs correlated with OSDI, and 16/26 correlated with conjunctival staining scores. Five upregulated DEGs (*CFB, CFI, IL1R1, IL2RG, IL4R*) were uniquely negatively correlated with TBUT. These data indicate that the conjunctiva of SS patients exhibits a phenotype of immune activation, although some genes could be inhibitory. Some of the DEGs and pathways overlap with previous DEGs in salivary gland biopsies, but new DEGs were identified, and some of these correlated with symptoms and signs of dry eye. Our results indicate that gene analysis of conjunctiva imprints is a powerful tool to understand the pathogenesis of SS and develop new therapeutic targets.

## Introduction

Patients with Sjögren syndrome (SS) develop an ocular surface disease called keratoconjunctivitis sicca (KCS). Pathological changes in the conjunctiva in KCS include altered epithelial differentiation with increased expression of cornified envelope precursors (1) that are found in the epidermis (involucrin, SPRR-2G) (2, 3) and dysfunction and loss of the goblet cells that secrete gel-forming mucin that is essential for maintaining tear film stability (4). Evidence from clinical and mouse model studies indicates innate, and adaptive immune mediators and cells contribute to these pathological changes based on clinical correlation with disease severity and improvement following topical treatment with the immunomodulatory agent cyclosporine A (5–8). Current methods to diagnose SS KCS include the use of dyes that stain conjunctival epithelial cells lacking a mucin coating or impression cytology to measure conjunctival goblet cell density, but they do not measure disease-relevant biomarkers. Additionally, the mechanism(s) by which this epithelial pathology develops is not entirely understood. An increased number of activated antigen-presenting cells (APCs) and increased interferon-γ expression have been found in the conjunctival epithelium in KCS (1, 6, 8–10). Interferon IFN-γ disrupts cholinergic mediated secretion and causes unfolded protein response (UPR) and apoptosis of conjunctival goblet cells (11, 12). Additional inflammatory mediators have been detected in the conjunctiva (1, 13, 14).

The purpose of this study was to compare the expression of immune-related molecular factors in the conjunctiva of normal subjects and SS KCS using the NanoString® Immunology panel.

## Methods

The study was conducted following the Declaration of Helsinki, and the Baylor College of Medicine Institutional Review Board approved the protocol and informed consent form before study initiation. Written informed consent was obtained from all participants after explaining the purpose and possible consequences of the study. Female control subjects and patients with SS were enrolled in the study from January 2019 to January 2021. SS patients were recruited from the multispecialty SS clinic at Baylor College of Medicine (BCM) and had a complete ocular, oral, and rheumatologic evaluation, including a panel of serum autoantibodies, and met proposed the American College of Rheumatology/European League Against Rheumatism diagnostic criteria for SS (15).

Symptom assessment in dry eye (SANDE) and Ocular Surface Disease Index (OSDI) symptom questionnaires, fluorescein tear break-up time (TBUT), Schirmer I test, cornea fluorescein and conjunctival lissamine green dye staining, and tear meniscus height (TMH) measurement using optical coherence tomography (OCT) were performed as previously described (16, 17). The ocular surface clinical parameters were all measured by the same observer (S.C.P.). Dry Eye Workshop (DEWS) criteria were used to grade clinical severity (18).

Healthy control subjects had no eye irritation, a TBUT ≥ 7 s, Schirmer 1 ≥ 10 mm, corneal fluorescein score ≤2, conjunctival lissamine score ≤ 2, and no meibomian gland disease. Subjects were excluded if they had prior laser-assisted *in situ* keratomileusis or corneal transplantation surgery, cataract surgery in the past year, punctal occlusion with plugs or cautery, a history of contact lens wear, use of topical medications other than preservative-free artificial tears, or chronic use of systemic medications known to reduce tear production. They were instructed not to instill any eye drops on the day of the evaluation.

All SS KCS patients had a severity score ≥of 3, and healthy controls had a severity score = 0.

### Conjunctival Impression Cytology and RNA Extraction

Cells were obtained by impression cytology of the temporal bulbar conjunctiva using the EyePrim™ device (Opia Tech, Paris, France) that applies a porous membrane with uniform pressure to the conjunctival surface. After collection, the membrane was placed in 0.5 mL RNA lysis buffer (Qiagen, Valencia, CA, USA) containing 1% 2-mercaptoethanol and immediately stored at -80°C. Total RNA was isolated using an RNeasy Mini Kit (Qiagen) following the manufacturer’s protocol. The RNA was eluted in 15 μL RNase-free water- The RNA concentration was measured by its absorption at 260 nm using a spectrophotometer (NanoDrop 2000; Thermo Scientific, Wilmington, DE, USA) and Agilent Bioanalyzer.

### NanoString^®^ and Data Analysis Using ROSALIND^®^ NanoSstring Gene Expression Methods

Five hundred ninety-four transcripts were quantified with the NanoString® nCounter multiplexed target platform using the Human Immunology V2 panel (www.nanostring.com). nCounts of mRNA transcripts were normalized using the geometric means of 15 housekeeping genes *(ABCF1, ALAS1, EEF1G, G6PD, GAPDH, GUSB, HPRT1, OAZ1, POLR1B, POLR2A, PPIA, RPL19, SDHA, TBP, TUBB*). Data were analyzed by ROSALIND^®^ (https://rosalind.onramp.bio/), with a HyperScale architecture developed by ROSALIND, Inc. (San Diego, CA). Read Distribution percentages, violin plots, identity heatmaps, and sample MDS plots were generated as part of the QC step. Normalization, fold changes, and p-values were calculated using criteria provided by NanoString^®^. ROSALIND^®^ follows the nCounter^®^ Advanced Analysis protocol of dividing counts within a lane using the same lane’s geometric mean of the normalizer probes. Housekeeping probes to be used for normalization are selected based on the geNorm algorithm as implemented in the NormqPCR R library (19). The abundance of various cell populations is calculated on ROSALIND using the NanoString® Cell Type Profiling Module. ROSALIND performs filtering of Cell Type Profiling results to include scores with a p-value lower than or equal to 0.05. Fold changes and P-values are calculated using the fast method described in the nCounter^®^ Advanced Analysis 2.0 User Manual. The Gene Set Analysis (GSA) module from NanoString® is incorporated into ROSALIND to summarize the global significance score and the directed global significance score. GSA summarizes the change in regulation within each defined gene set relative to the baseline, as described in the nCounter^®^ Advanced Analysis 2.0 User Manual. P-value adjustment is performed using the Benjamini-Hochberg method of estimating false discovery rates (FDR). Clustering genes for the final heatmap of differentially expressed genes was done using the PAM (Partitioning Around Medoids) method using the FPC R library (20) that considers the direction and type of all signals pathway the position, role, and type of every gene. Hypergeometric distribution was used to analyze the enrichment of pathways, gene ontology, domain structure, and other ontologies. The topGO R library (20) was used to determine local similarities and dependencies between GO terms to perform Elim pruning correction. Several database sources were referenced for enrichment analysis, including Interpro (21), NCBI5 (22), MSigDB (23, 24), REACTOME (25), WikiPathways (26). Enrichment was calculated relative to a set of background genes relevant for the experiment. Data analyzed in ROSALIND was downloaded, and Volcano plots of differential expression data were plotted using the −log10 (P-value) and log2 fold change using GraphPad Prism (Version 9, GraphPad Software, San Diego, CA). Heatmaps were also constructed using GraphPad Prism. Venn diagrams were made using Venny 2.1 (27). Over-represented pathway investigation was performed using www.innatedb.com (28).

### Statistical Analysis

Comparisons of OSDI, SANDE, TMH, TBUT, corneal and conjunctival scores between HC and SS subjects were performed using non-parametric Mann Whitney U test using GraphPad Prism version 9.0 (GraphPad Sofware). Non-parametric Spearman correlations using GraphPad Prism investigated the correlations of OSDI, TBUT, corneal and conjunctival score with up or down-regulated DEGs.

## Results

### Patient Demographics

Nineteen control subjects and seven age-and-sex-matched SS KCS patients were enrolled from January 2019 to January 2021 (**Table 1**). The control subjects had minimal eye irritation symptoms and no clinical signs of KCS, while SS KCS patients had significant symptoms and clinical signs. Statistical comparison of OSDI and SANDE scores, tear meniscus height (TMS), tear-break-up time (TBUT), corneal and conjunctival staining scores between controls and SS KCS are shown in **Table 2**.

**Table 1:** Demographic Characteristics of Study Groups.

	N, subjects	Age, mean, years	Age, range, years	Female/Males
Healthy controls	19	57	41-76	19/0
SS-KCS	7	58	40-70	7/0

**Table 2:** Summary of clinical data, showing mean ± standard deviation.

	OSDI, score	SANDE, score	Tear meniscus height, μm	Tear-break-up time, seconds	Corneal staining score*	Conjunctival staining score^†^
Healthy controls	9.2 ± 8.4	13.5 ± 17.7	419.1 ± 197	9.5 ± 2.5	0.1 ± 0.32	0.05 ± 0.23
SS-KCS	58 ± 11	88.6 ± 10.6	223.4 ± 154	2.7 ± 0.9	7.4 ± 3.0	5.6 ± 1
P value**	P<0.0001	P<0.0001	P<0.05	P<0.0001	P<0.0001	P<0.0001

*Corneal fluorescein dye staining.

^†^Conjunctival lissamine green dye staining.

OSDI, Ocular Surface Disease Index questionnaire.

SANDE, Symptom Assessment iN Dry Eye (visual analog questionnaire).

**P values were calculated using the Mann-Whitney U Test.

### Gene Expression Analysis in the Conjunctiva of SS KCS Identifies Upregulated Targets

**Figure 1:**
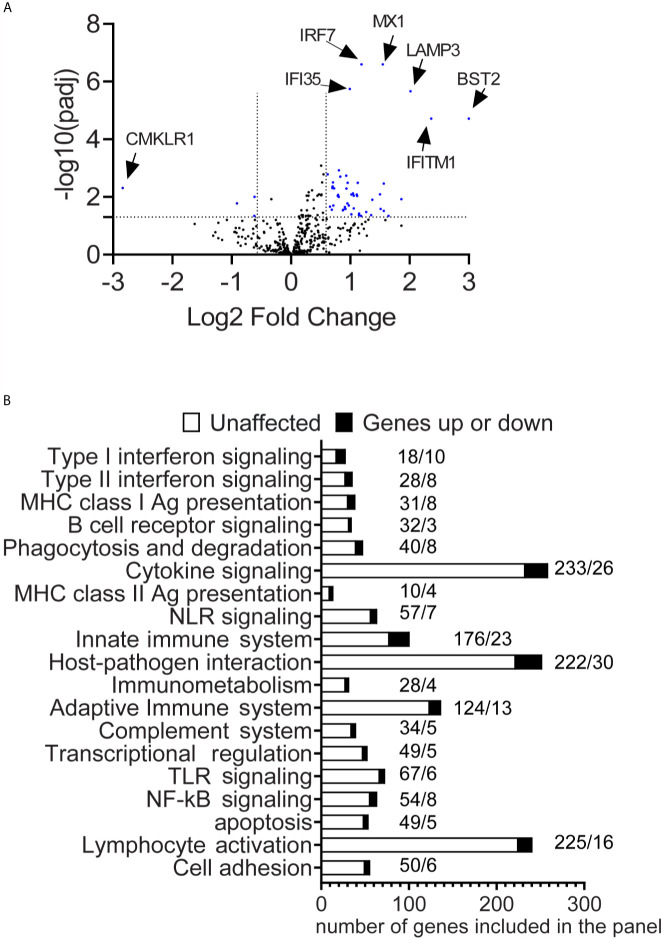
Differential expression of immune genes in the conjunctiva of SS patients. **(A)** A volcano plot shows −log10(p-value) and Log 2-fold change in gene expression in SS-KCS patients compared to control subjects (fold increase >1.5 or fold decrease <1.5 and p adjusted value of 0.05). P-value thresholds were adjusted using the Benjamini–Hochberg method of estimating false discovery rates. The horizontal line indicates p=0.05, and the two vertical lines indicate greater or lower than 1.5-fold. **(B)** Pathway descriptors are included in the panel. Dark bars indicate modulated genes (up or down), and white bars show unaffected genes. SS-KCS, Sjögren Syndrome keratoconjunctivitis sicca. Numbers above the bars indicate the number of unaffected genes (left) followed by the affected genes (right).

The conjunctiva of SS KCS shows epithelial metaplasia, goblet cell loss, T cell infiltration, and increased expression of *HLA-DR* and *IFNG* mRNA (6, 10, 29–32). We and others have shown that impression cytology of the conjunctiva collects a mix of epithelial and immune cells (8, 14, 33). To gain insights into the molecular mechanisms involved locally, impression cytology of the conjunctiva was used to collect conjunctival cells, which were then lysed and processed for RNA analysis using a NanoString® nCounter panel enriched for immune genes (Immunology V2). This panel allows for the simultaneous evaluation of 594 genes. Gene expression analysis was quantified in the conjunctiva of healthy and SS KCS patients using nCounter and ROSALIND software, as described in methods. Out of the 594 genes, eight genes were not detected; 53 were significantly modulated in SS KCS patients: 4 genes were downregulated (*TRAF5, TGFBI, KLRAP1*, and *CMKLRI)*, and 49 genes were upregulated using a stringent 1.5-fold increase or decrease threshold (volcano plot in **Figure 1A**). Significance was plotted against fold change (log2 values) using a -log10 of the adjusted p-value for each dot. The top 20 upregulated differentially expressed genes (DEGs) in descending order were *BST2, IFITM1, LAMP3, CXCL1, IL19, CFB, LY96, MX1, IL4R, CDKN1A, SERPING1, HLA-DRB3, S100A8, IRF7, ICAM1, C4A/B, TNFAIP3, CD74, HLA-B*, and *TAP1* (**Table 3**). A complete list of DEGs can be found in **Supplementary Table 1**.

**Table 3:** Top 20 upregulated and 4 downregulated genes in SS KCS compared to controls.

	Gene	Fold change	P adjusted value
Up	BST2	7.99701	1.93E-05
	IFITM1	5.15578	1.93E-05
	LAMP3	4.04506	2.16E-06
	CXCL1	3.63536	0.011922
	IL19	3.12475	0.045266
	CFB	2.95921	0.003435
	LY96	2.95703	0.030392
	MX1	2.92781	2.52E-07
	IL4R	2.84982	0.026096
	CDKN1A	2.833	0.008089
	SERPING1	2.58668	0.012468
	HLA-DRB3	2.5539	0.042507
	S100A8	2.40634	0.033422
	IRF7	2.28104	2.52E-07
	ICAM1	2.26976	0.045266
	C4A/B	2.21685	0.038018
	TNFAIP3	2.21593	0.042507
	CD74	2.21036	0.042507
	HLA-B	2.18027	0.003229
	TAP1	2.16574	0.009172
	STAT2	1.53471	0.001635
Down	KLRAP1	-1.53118	0.00998
	TRAF5	-1.53613	0.045266
	TGFBI	-1.88146	0.016735
	CMKLR1	-7.14587	0.004898

### Differential Pathways Are Activated in the Conjunctiva of SS KCS Patients

**Figure 2:**
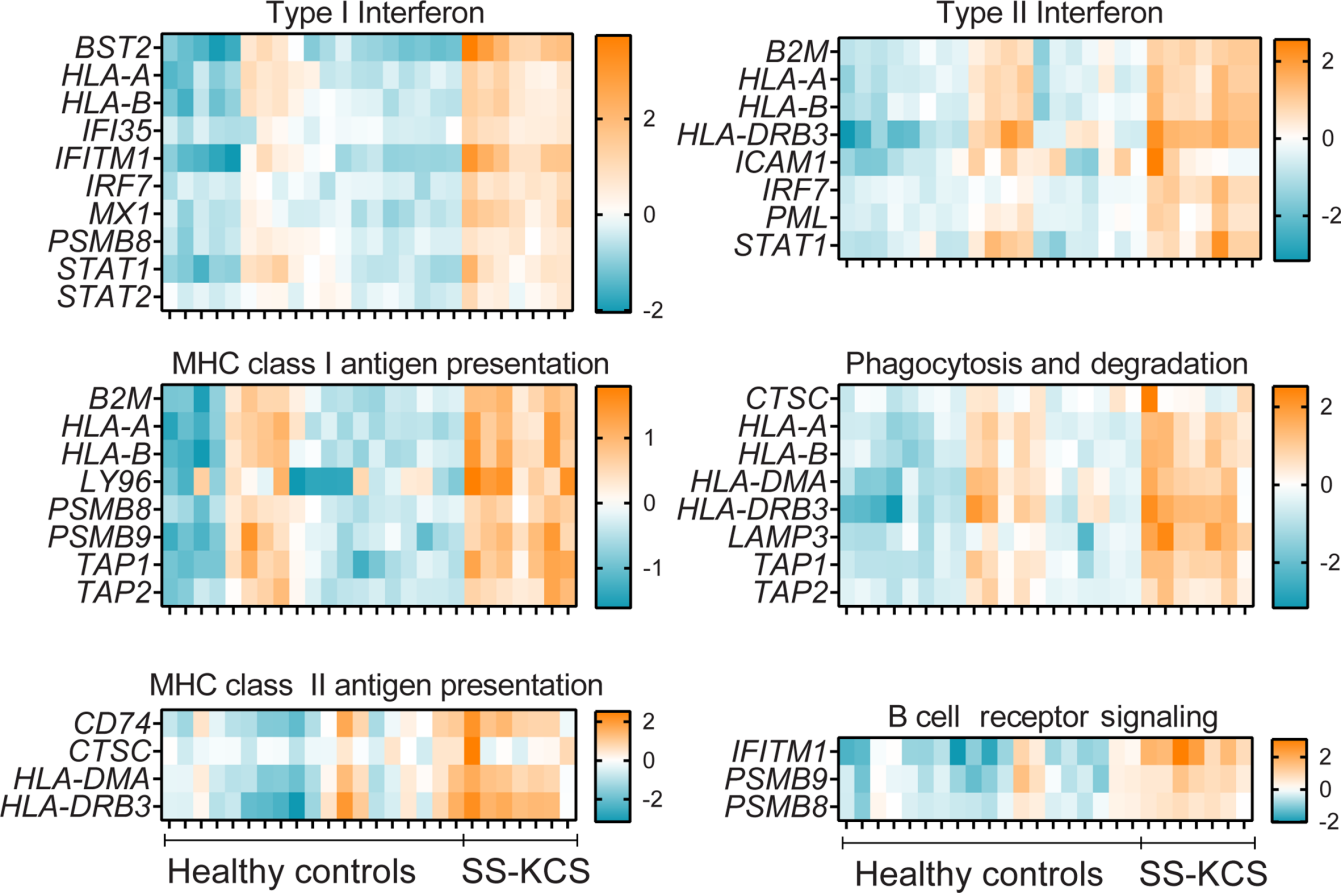
A heatmap of expression of immune pathways that were upregulated in SS KCS conjunctiva. Heatmaps show Type I and II Interferon, MHC class I and II antigen presentation, phagocytosis and degradation, and B cell receptor signaling pathways.

**Figure 3:**
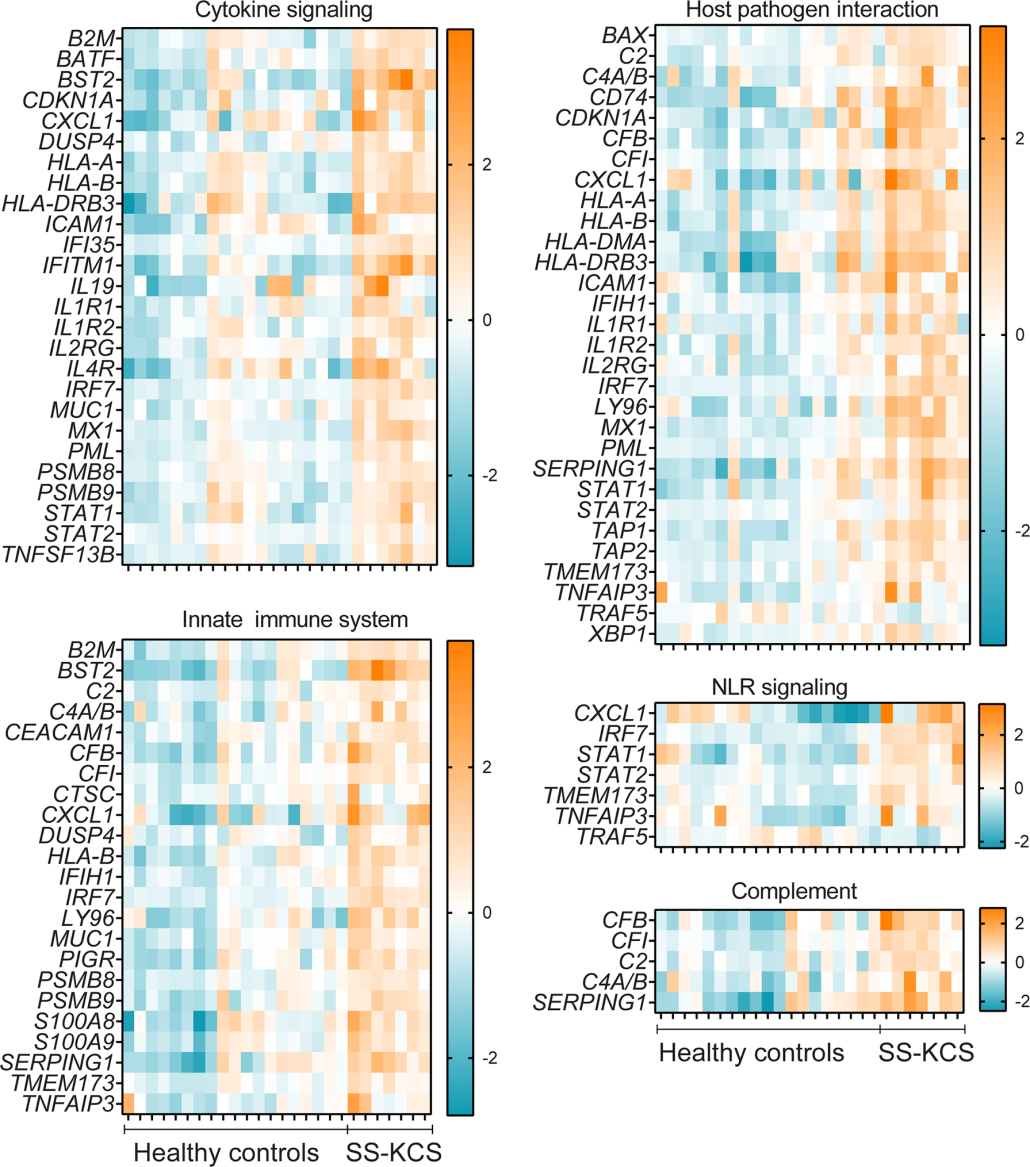
Heatmaps of expression of immune pathways with significant genes upregulated or downregulated gene (*TRAF5*) in SS KCS conjunctiva. Heatmaps show Cytokine signaling, Host-pathogen interaction, Innate immune system, Nod-like-receptor (NLR) signaling, and Complement pathways.

**Figure 4:**
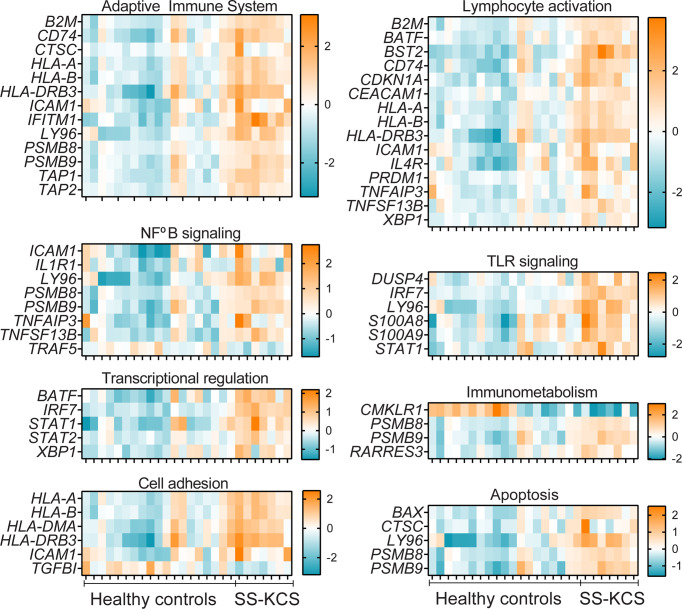
Heatmaps of Adaptive immune system, Lymphocyte activation, NFkB, TLR signaling, Transcriptional regulation, Immunometabolism, Cell adhesion, and apoptosis. The downregulated genes *TRAF5, CMKLR1*, and *TGFBI*, are found in the NFkB signaling, Immunometabolism, and Cell adhesion pathways.

Previous studies have identified a significant role for inflammation in the conjunctiva of SS KCS patients (6, 13, 14, 34). To gain insight into the pathways involved, we analyzed which DEGs have been annotated or identified in immune pathways using the ROSALIND software and nCounter software, using the gene set analysis. This algorithm calculates a T-statistic for each gene against each covariate in the model. A gene set’s global significance score for a covariate measures the cumulative evidence for the differential expression of specific genes in a pathway. Out of the 32 immune pathways included in the panel, the top 20 pathways are shown in **Figure 1B** (global significance range from 2.0 to 4.24). For each pathway, modulated genes and unaffected genes are shown. The pathways (**Figures 2**–**4**) and their respective global significance scores are Type I Interferonsignaling (4.24), Type II Interferon signaling (2.91), MHC Class I antigen presentation (2.84), B cell receptor signaling (2.44),Phagocytosis and degradation (2.44), Cytokine signaling (2.40), MHC Class II antigen presentation (2.37), NLR signaling (2.36), Innate immune system (2.32), Host-pathogen interaction (2.31), Immunometabolism (2.28), Adaptive immune system (2.28), Complement system (2.25), Transcriptional regulation (2.24), TLR signaling (2.23), NFkB signaling (2.23), Apoptosis (2.15), Lymphocyte activation (2). As expected, not all pathways in the panel were identified in our results.

Some DEGs overlapped in more than one pathway. For example, the proteasome subunits beta type-8 *(PSMB8)* and type-9 *(PSMB9)* were annotated in multiple pathways (type I interferon, MHC Class I antigen presentation, B cell receptor signaling, cytokine signaling, innate immune system, adaptive immune system, NFkB, immunometabolism, and apoptosis). Both PSMB8 and PSMB9 are integral parts of the immunoproteasome and, together with TAP1 and TAP2, are involved in the MHC class I presentation. TAP1 and TAP2 are transporters (-1,-2, respectively) and ATP binding cassette subfamily B members. Similar to *PSMB8* and *PSMB9*, *TAP1* and *TAP2* were also annotated in multiple pathways (MHC class I presentation, phagocytosis, host-pathogen interaction, adaptive immune response, **Figures 2** **–** **4**). However, other DEGs were uniquely annotated. An example is *LAMP3* (lysosome-associated membrane glycoprotein 3 or DC-LAMP), which was uniquely annotated in the phagocytosis pathway.

It has been demonstrated that SS patients have a Type I and II Interferon signature in their serum (35–38). Accordingly, we observed many DEGs involved in Type I and II interferon responses in the conjunctiva, such as *BST2*, *IFITM1, IRF7*, and *MX1* (**Figure 2**). *BST2* (*bone marrow stromal antigen 2*) was the most upregulated DEG in our results. Elevated *BST2* levels are found in labial gland SS biopsies compared to controls and these levels correlated with rheumatoid factor and B2M serum levels (39)*. IFITM1* (interferon-induced transmembrane protein 1), *IRF7* (interferon regulatory factor 7), and *MX1* (interferon-induced GTP-binding protein Mx1) are also interferon-responsive genes (40) that were upregulated with greater significance in our results. *IFITM1* was the second most upregulated DEGs in our results, and it is involved in Type I interferon and B cell receptor signaling, which also showed upregulation of *PSMB8* and *PSMB9.*

The complement system participates in the lysis of infectious organisms by facilitating the binding of antibodies and phagocytic cells to microorganism membranes. It also promotes inflammation and immune clearance. There were five upregulated DEGs in our results, as shown in **Figure 3** (*CFB, CFI, C2, C4A/B,* and *SERPING1)*. CFB, a component of the alternative pathway of complement, is also increased in cerebrospinal fluid of patients with SS and fatigue (41). Both C2, C4A, and C4B proteins are known to participate in the generation of the classical complement pathway. The complement pathway is also part of the innate immune system, which showed many DEGs, such as *CEACAM-1, CXCL1, S100A8, S100A9, PIGR*, and *MUC1*. *CEACAM-1* (carcinoembryonic antigen-related cell adhesion molecule 1) is a co-inhibitory molecule in mucosal immunology (42). CXCL1 is an antimicrobial protein that is a chemoattractant for neutrophils, which have been implicated in dry eye (43, 44). *PIGR* is a polymeric immunoglobulin receptor (also known as secretory component) that mediates polymeric IgA and IgM transport across mucosal epithelial cells. It is highly expressed in the lacrimal gland and tears (45) and, once secreted, can act as an antimicrobial agent on its own (46).

The involvement of the major inflammatory pathway NFkB in SS is well established in the literature (47, 48). **Figure 3** shows the DEGs involved in this pathway: *ICAM-1, IL1R1, Ly96, TNFAIP3* (encoding tumor necrosis factor, alpha-induced protein 3 or A20 protein), *TNFSF13B*, and downregulation of *TRAF5*. *TNFSF13B* encodes BAFF, B cell activation factor, a protein involved in B cell survival. BAFF SNPs and mutations have been shown in SS patients (49, 50). *TRAF5*, TNF receptor-associated factor 5, is a scavenger protein involved in TNF and NFkB activation and mediates IL-17-ACT1 interactions. *TRAF5* SNPs showed an association with rheumatoid arthritis compared to control subjects (51). Genome-wide association studies have shown that *TNFA1P3* is upregulated in Chinese SS patients (52). *TNFAIP3*, *HLA-DMA*, *HLA-DR* genes (*HLA-DRB5* and *HLA-DRB1*), *CDKN1B* (cyclin-dependent kinase inhibitor 1B), *IFITM1, NKFB1* (nuclear factor of kappa light polypeptide gene enhancer in B-cells 1), and *IRF7* also elevated in labial salivary biopsies (53). Other small molecules and drugs that target the NFkB pathway in SS are undergoing clinical trials [reviewed in (54)].

Five transcription factors were upregulated and are shown in **Figure 4**: *BATF, IRF7, STAT1, STAT2, XBP1*. *BATF* (basic leucine zipper transcription factor, ATF-like**)** and is involved in Th17 differentiation. *BATF* ^-/-^ mice have decreased Th17 and ameliorated experimental autoimmune encephalitis (55), a model where Th17 cells are pathogenic. IL-17 has been implicated in dry eye and models of SS (56–61). IRF7 is a transcription factor that mediates IFN-α signaling. IRF7 has been found elevated in the SS patients, where it is thought to mediate the IFN signature (35–38, 62).

The immunometabolism pathway had two DEGS*; CMKLR1* and *RARRES3 (-7.1- and 1.6-fold* change, respectively, **Figure 4**). *CMKLR1* encodes chemerin chemokine-like receptor 1, also known as resolvin E1 receptor, a critical molecule in inflammation resolution (63). A CMKLR1 agonist antibody has shown promise in pre-clinical models of intestinal mucosal inflammation (64). *RARRES3*, retinoic acid receptor responder protein 3, is an intracellular retinoid receptor responder that belongs to RNA pattern recognition receptors. RARRES3 has been shown to interact with the immunoproteasome and upregulate IRF1 in breast cancer cells, and to stimulate the proliferation of keratinocytes (65).

*TGBI* and *ICAM-1* were differentially modulated in our dataset and are annotated in the cell adhesion pathway (down and upregulated DEGs, respectively, **Figure 4**). Mutations of *TGFBI (*transforming growth factor beta-induced) have been implicated in cornea dystrophies (66). *ICAM-1*, intercellular adhesion molecule-1, is a critical molecule in the adaptive immune system that facilitates cell adhesion and promotes cell adhesion. ICAM-1 is elevated in SS serum and mouse models of dry eye disease (67–70). Lifitegrast, which blocks the immunological synapse of lymphocyte function-associated antigen/ICAM-1, is an FDA-approved eye drop for dry eye disease (71–73).

Pathway overrepresentation analysis using the InnateDB tool (28) showed that the “Antigen processing and presentation” pathway was over-represented in our results (10% of genes, P<0.00001, p-adjusted value), using the Kyoto Encyclopedia of Genes and Genomes (KEGG) database. Genes involved in this pathway were *B2M, CD74, HLA-A, HLA-B, HLA-DMA, TAP1,* and *TAP2.* These same genes were annotated in Type I and II interferon responses, MHC class I and II antigen presentation, phagocytosis, and degradation in the NanoString® pathways (**Figure 2**). *B2M* (beta-2 microglobulin), involved in MHC Class I antigen presentation to CD8 T cells, has shown to be a hub for protein interaction in SS (74). HLA-A and HLA-B are major histocompatibility complexes that form a heterodimer with B2M.

These results demonstrate the pleiotropic abilities of specific genes, which participate in multiple pathways.

### Correlation of Upregulated DEGs With Symptoms Severity Score and Signs

We performed Spearman correlation analysis between the normalized counts *vs.* OSDI, or TBUT, or corneal staining score, or conjunctival score to investigate which DEGs correlated with symptoms and signs of dry eye. No correlations between gene normalized counts and corneal fluorescein staining scores were observed in any of the genes in our results.

**Figure 5:**
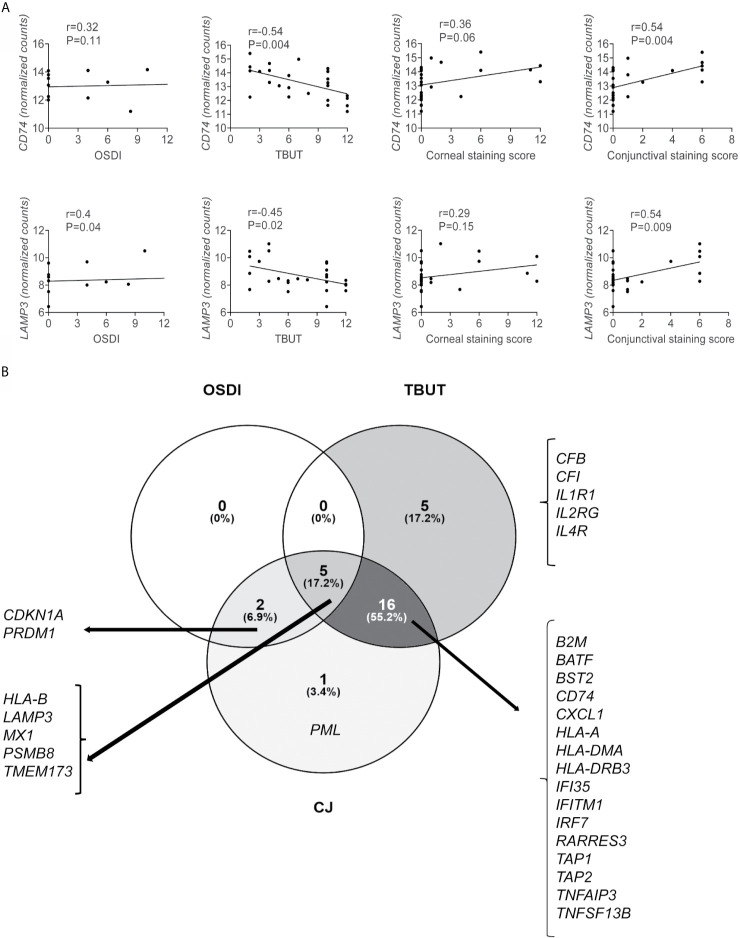
A subset of upregulated genes correlates with ocular symptoms and signs. Spearman correlations of clinical symptoms (OSDI), Tear-break-up time (TBUT), and conjunctival (CJ) staining score with up or downregulated DEGs were calculated using GraphPad Prism software using clinical data from all subjects (19 healthy controls and 7 SS KCS subjects). **(A)** Representative graph showing Spearman correlations of *CD74* and *LAMP3 vs.* OSDI, TBUT, corneal staining score, and conjunctival staining score. Additional genes are shown in **Table 3**. **(B)** Venn diagram showing common and unique correlation of genes and OSDI, TBUT, and conjunctival staining score. R = coefficient of correlation. Please note that GraphPad Prism does not plot all dots if they overlap.

From the 54 DEGs, 29 DEGs correlated with either OSDI, TBUT, or conjunctival staining score (**Table 4**), and some DEGs correlated with more than one clinical sign. Only upregulated DEGs showed any significant correlation. Two representatives Spearman correlation graphs are shown in **Figure 5A**: 1) *CD74*, a critical step in MHC II pathway (75), inversely correlated with TBUT and positively with conjunctival staining score, 2) *LAMP3*, a lysosomal protein involved in autophagy (76), which correlated with OSDI, TBUT, and conjunctival staining score. Increased levels of CD74 have been previously reported in minor salivary gland SS biopsies (62, 77).

**Table 4:** Spearman correlations with clinical symptoms (OSDI), Tear-break-up time (TBUT), and conjunctival (CJ) staining score were calculated using GraphPad Prism software using clinical data from all subjects (19 healthy controls and 7 SS KCS subjects). Out of 54 DEGs, only 29 of the upregulated DEGs showed a correlation.

Genes	OSDI	TBUT	CJ staining score	Pathways
CDKN1A	0.4*		0.48*	LA, HPI, CS
PRDM1	0.38*		0.39*	LA
HLA-B	0.37*	-0.46*	0.45*	TI, TII, MHC I, P, AIM and CA
LAMP3	0.4*	-0.45*	0.5*	P
MX1	0.43*	-0.46**	0.5**	TI, CS, HP
PSMB8	0.39*	-0.49**	0.5**	TI, MHC I, CS, IMS, AIM, NFkB, IMM, and Apo
TMEM173	0.39*	-0.49**	0.38*	HPI, IIS, NLR
CFB		-0.38*		HPI, IMS, C
CFI		-0.38*		HPI, IMS, C
IL1R1		-0.45*		CS, HPI, NFkB,
IL2RG		-0.4*		CS, HPI
IL4R		-0.4*		CS
B2M		-0.5*	0.45*	MHC I, TII, CS, IMS and AIM, LA
BATF		-0.46*	0.39*	CS, LA
BST2		-0.4*	0.43*	TI, MHC II, CS, IIS
CD74		-0.54**	0.54**	MHC II, HPI, AIS, LA
CXCL1		-0.42*	0.41*	CS, HPI, IIS, NLR
HLA-A		-0.43*	0.47*	TI, TII, MHC I, P, CS, HPI, AIS, LA, CA
HLA-DMA		-0.5**	0.51**	MHC II, P, HPI, CA
HLA-DRB3		-0.44*	0.5*	TII, P, CS, HPI, AIS, LA, CA
IFI35		-0.44*	0.44*	TI, CS
IFITM1		-0.47*	0.45*	TI, CS, AIS
IRF7		-0.4*	0.45*	TI, TII, CS, HPI, IIS, NLR, TLR, TS
RARRES3		-0.4*	0.41*	IMM
TAP1		-0.48*	0.5*	MHC I, P, HPI, AIM
TAP2		-0.39*	0.44*	MHC I, P, HPI, AIS
TNFAIP3		-0.4*	0.38*	HPI, IIS, NLR, LA, NFkB
TNFSF13B		-0.37*	0.38*	CS, LA, NFkB
PML			0.42*	TII, CS, HPI

AIS, adaptive immune system; Apo, Apoptosis; C, complement; CA, cell adhesion; CS, cytokine signaling; HP, Host-pathogen interaction; IMM, immunometabolism; IIS, Innate Immune system; LA, Lymphocyte activation; MHC I, MHC Class I antigen response; MHC II, MHC Class II antigen presentation; NFkB, NFkB signaling; P, Phagocytosis; NLR, Nod-like-receptor signaling; TLR, toll-like-receptor signaling; TS, transcriptional signaling; TI, Type I interferon, TII, Type II interferon. Numbers indicate the coefficient of correlation. *P<0.05, **P<0.01.

Next, we used the data in **Table 4** to make Venn diagrams to identify the unique and shared correlations (**Figure 5B**). We identified that 26/29 DEGs showed an inverse correlation with TBUT; 5 of these 26 DEGs showed a positive correlation with OSDI, and 16/26 showed a positive correlation with conjunctival staining score. Five upregulated DEGs (*CFB, CFI, IL1R1, IL2RG, IL4R*) were uniquely inversely correlated with TBUT, while *PML* (promyelocytic leukemia protein) was uniquely correlated with conjunctival staining score (**Table 4**). PML is upregulated by STAT1 and participates in TNF-α and IFN-α induction of angiogenesis (78). Detection of PML in liver biopsies has been used as a diagnostic tool for autoimmune liver diseases, including primary biliary cirrhosis (79, 80). In addition to *LAMP3*, only an additional 6 DEGs correlated with OSDI (*CDKN1A, PRDM1, HLA-B, MX1, PSMB8*, and *TMEM173)*. The disconnection between symptoms and signs in SS KCS is well described in the literature.

## Discussion

The pathogenesis of SS has been a subject of intense investigation. The cornea and the conjunctiva develop pathological changes in SS KCS (81). Because it is not possible to obtain cells from the cornea noninvasively, we performed multiplex gene analysis of cells obtained from the conjunctiva using impression cytology using an Immunology panel. Impression cytology of conjunctiva has been used to investigate conjunctival goblet cell density, epithelial metaplasia, and obtain cells for flow cytometry (10, 82–87). It has also been used to investigate gene expression between different forms of dry eye disease and comparisons between healthy controls and SS KCS, as in our studies (6, 13, 14).

A careful investigation of pathways in our immunology panel confirmed some findings in literature but also unraveled new pathways and DEGs that had not been previously associated with SS. Pathways that have been previously associated with SS include Type I and Type II Interferon, MHC Class I and II presentation, Innate immune system, Phagocytosis, NFkB, B cell receptor to name a few. Increased expression of the interferon-inducible genes is consistent with our findings of increased IFN-γ expression in the conjunctiva of patients with SS KCS (1, 6) and that interferon-γ produced by NK and T cells increases chemokine expression and promotes goblet cell loss in conjunctival of mouse SS models (11, 88–92). Interestingly, the B cell receptor signaling pathway was upregulated in our results involving the conjunctiva. The involvement of B cells in SS is well established, as SS patients have B cell infiltration in salivary and lacrimal glands in humans and rodents. SS patients have an increased risk for non-Hodgkin lymphomas (93–96). The role of B cell receptor signaling in the conjunctiva of SS KCS patients has not been thoroughly investigated.

There were several upregulated genes in the host-pathogen, NLR signaling, and TLR signaling pathways. While no ocular surface pathogen has been identified in the pathogenesis of SS, there is a link between intestinal dysbiosis (with increased proteobacteria genera) and the severity of SS KCS (97, 98). Additionally, antibiotic-induced dysbiosis in mice increased the ocular surface inflammatory response to topically applied lipopolysaccharide (99) and led to a worse dry eye phenotype after desiccating stress (97). Upregulated genes in these pathways can heighten the TLR signaling (e.g., *LY96*) and innate and adaptive immune responses to microbial products that can be released into the circulation with intestinal dysbiosis. Several of the upregulated DEGs (*IL-4R, IL-19*, and *DUSP*) may have compensatory immunosuppressive functions. Th2 cytokines, such as IL-4 and IL-13, are usually implicated in allergic conjunctival inflammation (100), and IL-13 signaling through its heterodimeric receptor, which includes the IL-4R, stimulates goblet cell proliferation and mucin production in the conjunctiva (101, 102). Similarly, IL-19 is an anti-inflammatory cytokine that belongs to the IL-10 family and participates in the modulation of macrophage from M1 to M2, and DUSP4 negatively regulates phosphorylation of several MAPKs (103–105).

One pathway that was discovered in our panel was immunometabolism, with two unique DEGs (*CMKLR1* and *RARRES3). CMLKR1* also participates in the resolution of inflammation. Topically applied resolvin E1 analogs improved corneal staining, goblet cell density, and improved tear secretion while decreasing CD11b^+^ and CD4^+^ infiltration to the cornea in a murine model of dry eye disease (106, 107). A protective role for resolvin D has been shown in the salivary gland of SS animal models (108). Interestingly, *RARRES1*, another member of the same family, is elevated in SS labial salivary gland biopsy microarray studies (53). This is the first time that RARRES3 is associated with the conjunctiva of SS KCS patients.

Our results showing increased *XBP-1* and *STAT1* levels agree with the literature and with our previous publication that showed increased *XBP-1* and *GRP78* in the SS KCS conjunctiva compared to healthy controls (11). Increased levels of STAT1 have been previously reported in humans and animal models, also in the conjunctiva (13, 14, 62, 109). *XBP-1* (X-box binding protein-1) participates in ER stress and UPR, decreasing salivary gland protein secretion (110). In the conjunctiva, XBP-1 participates in the IFN-γ-induced UPR by decreasing Muc5ac protein secretion from cultured conjunctival goblet cells (11).

Correlation analysis of the DEGs with clinical signs identified that 53% of DEGs correlated with at least one clinical sign. IFITM1, the second most upregulated DEG in our results, correlated with TBUT and conjunctival staining score. Elevated IFITM1 levels have been reported in the conjunctiva of dry eye patients (111). PSMB8 correlated with OSDI, TBUT, and conjunctival staining, while TAP1 and TAP2 correlated with TBUT and conjunctival staining. Epigenetic changes in PSMB8 and TAP1 have been reported in human labial gland biopsies in SS patients from the SICCA repository (112). Increased mRNA and protein expression in PSMB8 and PSMB9 in epithelial and immune cells of salivary glands have been reported (77, 113). LAMP3 was one of the only five DEGs correlated with three ocular parameters (OSDI, TBUT, and conjunctival staining score). LAMP3 is a marker of mature DCs in humans (114), and we and others have shown that the conjunctiva of SS KCS patients is rich in antigen-presenting cells (8, 33, 115, 116). LAMP3+ patients have increased levels of autoantibodies in their serum, and *in-vitro*, overexpression of LAMP3 induces apoptosis of minor salivary gland epithelial cells (117). Adenovirus transfection with LAMP3 in mice induces an SS-like phenotype accompanied by decreased salivary function (118). CDKN1A, cyclin-dependent kinase inhibitor 1, p21, correlated with OSDI and conjunctival staining score. Elevated senescence markers CDKN1A (119) and CDKN1B have been found in minor salivary gland SS biopsies (62). Genetic deletion of p21 in lupus-prone mice promoted apoptosis of long-lived T and B cells, decreasing autoimmunity (120). Our results are the first to show elevated CDKN1A, PSMB8, and PSMB9 in the conjunctiva of SS patients.

Our studies agree with Kessal and colleagues, who reported increased *C2* and *CFB* (complement factor B) in the conjunctiva of SS KCS patients (13, 14). The complement system is activated at the ocular surface at low levels, where it is thought to participate in immunosurveillance (121). However, functional studies of the complement system in SS are primarily concentrated in the serum, where decreased complement levels have been found (122). Variants in *C4* genes (which segregate with *MHC II* genes) increase the odds ratio of SS (123). A systematic review in the literature showed that a deficiency of C1-inhibitor (encoded by *SERPING1)* was present in autoimmune diseases (124). Our study adds *CFI* (*complement factor I*), *C4/B*, and *SERPING1* to the list of upregulated genes in the complement cascade in the conjunctiva of SS KCS patients. The role of the complement cascade in the conjunctiva warrants further investigation.

Antigen presentation and processing, including MHC class I and class II pathways, were overrepresented in our results. Elevated *HLA-A, HLA-B, HLA-DMA* levels, together with other *HLA-DR* genes (*HLA-DRB5* and *HLA-DRB1*), have been found in labial salivary biopsies in SS patients (53, 77). Genome-wide studies have shown that DEGs *TAP1, TAP2, PSMB9, HLA-DR, HLA-DMA* have been associated with SS in more than one study (52, 125, 126). *LAMP3* was the third most upregulated DEG in our results, in agreement with previous studies that also reported elevated LAMP3 levels in SS (62, 117). *MX1* has also been found elevated in other cohorts of SS patients, including conjunctiva (13, 14), labial salivary biopsies (53, 62, 127, 128), and serum (129). When comparing our DEGs with previous studies that performed comprehensive gene analysis of the conjunctiva, our studies identified four overlapping DEGs (*MX1, C2, CFB, STAT1, STAT2*) (13, 14). Compared to studies that performed gene analysis in labial gland biopsies from SS patients, our results identified some overlapping DEGs (62, 74, 77, 113, 127, 128). Unique DEGs not previously associated with SS were *HLA-DRB3, CFI, CEACAM1*, *CXCL1, DUSP4, IL19, IL1R1, MUC1, SERPING1, S100A8, S100A9, PIGR, XBP1, although some proteins encoded by these DEGs have been associated with dry eye [XBP1, HL-A-DR* (11, 33, 115)*].* The majority of these DEGs participate in Innate immune response. Some of these proteins are secreted by epithelial cells and demonstrate the importance of the epithelial barrier in autoimmunity (130). Elevated levels of PIGR have been found in the SS saliva (131). MUC1, mucin 1, has been shown to regulate inflammation and TLR signaling after infections in mucosal sites (132). SNPs in the *MUC1* gene have been identified in SS and dry eye subjects (133–135). Interestingly, 5/50 upregulated genes in the conjunctiva of a mouse model of SS were also included in our upregulated DEGs [*MX1, ICAM1, S100A9, IRF7, BATF*, (136)].

The strengths of this study include robust gene expression data *via* NanoString and comprehensive measures of signs and symptoms of dry eye disease to allow correlation of gene expression levels with different objective measures of the disease. The limitations of our study include the relatively limited set of genes evaluated by this NanoString® platform and the restriction to immune-related genes as compared to the more open approach of RNA sequencing, though the use of the NanoString platform allowed for a direct focus on immune-related genes, which was the purpose of the study.

Further studies are necessary to validate these DEGs identified in our study, which identified shared and unique DEGs and pathways in the conjunctiva of SS KCS patients. These findings highlight that some therapies targeting immune mediators might be efficacious for SS KCS. The ocular surface also has unique activated pathways that warrant further investigation if these DEGs can be used as valid biomarkers modulated by therapies such as cyclosporine A and short-term steroids. Investigation of immune pathways in the conjunctiva might yield novel therapeutic targets for SS KCS.

## Data Availability Statement

The original contributions presented in the study are included in the article/**Supplementary Material**. Further inquiries can be directed to the corresponding author.

## Ethics Statement

The studies involving human participants were reviewed and approved by Baylor College of Medicine Institutional Review Board. The patients/participants provided their written informed consent to participate in this study.

## Author Contributions

CP, CT-V, LS, SP, ZY, and RB were involved in the conception and design of the study. CP, CT-V, LS, SP, and ZY were involved in data acquisition. CP, CT-V, LS, SCP, ZY, and RB were involved in data analysis and interpretation. CP drafted the manuscript. All authors contributed to the article and approved the submitted version.

## Funding

This work was supported by NIH EY026893 (CP); NIH EY002520 (Core Grant for Vision Research Department of Ophthalmology); BCM Genomic & RNA Profiling Core GARP Core [P30 Digestive Disease Center Support Grant (NIDDKDK56338) and P30 Cancer Center Support Grant (NCICA125123), NIH S10 grant (1S10OD02346901)]. Further research support was provided by Research to Prevent Blindness Stein Award (RB), Research to Prevent Blindness (unrestricted grant to the Dept. Of Ophthalmology), The Hamill Foundation, and The Sid Richardson Foundation. Claudia M. Trujillo-Vargas received supplemental salary support from Facultad de Medicina, Universidad de Antioquia, UdeA, Medellin, Colombia.

## Conflict of Interest

The authors declare that the research was conducted in the absence of any commercial or financial relationships that could be construed as a potential conflict of interest.
